# Role of Systemic Immune-Inflammatory Index and WBC Differential in Predicting the Severity of Acute Pulmonary Embolism: A Single-Center Observational Study at King Fahad Specialist Hospital, Buraydah, Saudi Arabia

**DOI:** 10.7759/cureus.110836

**Published:** 2026-06-14

**Authors:** Raana Zeeshan, Essam A Altowyan, Nawaf H Alharbi, Omar K Alsaawi, Rayan M Alharbi, Khalid S Alrashdi, Yousef F Alosaimi

**Affiliations:** 1 Hematology, King Fahad Specialist Hospital, Buraydah, SAU; 2 Internal Medicine, King Fahad Specialist Hospital, Buraydah, SAU; 3 Hematology, King Fahad Medical City, Riyadh, SAU

**Keywords:** acute pulmonary embolism, king fahad specialist hospital, saudia arabia, sii, wbc

## Abstract

Background

Early identification of high-risk pulmonary embolism (PE) is crucial for improving patient outcomes. The systemic immune‑inflammatory index (SII) has emerged as a potential biomarker of thromboinflammatory activity. This study aimed to assess the roles of SII and white blood cell (WBC) differentiation in predicting the severity of acute PE (APE).

Methods

This was a retrospective study of patients with APE admitted to King Fahad Specialist Hospital in Saudi Arabia from 2022 to 2025. Data included demographics, chronic diseases, and clinical features. Furthermore, vital signs, oxygen saturation, complete blood count with differential, echocardiography, and computed tomography angiography results at admission, and mortality rate were collected. All patients were classified according to severity using the Pulmonary Embolism Severity Index (PESI) score and PE risk stratification as massive, submassive, or non-massive. All statistical analyses were performed using IBM SPSS Statistics for Windows, version 26 (IBM Corp., Armonk, New York, United States).

Results

Among the 166 patients with APE (mean age: 57.1 years) included in this study, 93 patients (56%) were female, and hypertension and diabetes were the most common comorbidities, with 73 (44%) and 61 (36.7%) having them, respectively. Levels of inflammatory markers (WBC and neutrophils) and cardiac biomarkers (troponin) are markedly elevated in patients with massive PE. Receiver operating characteristic analysis showed that neutrophil count (area under the curve: 0.766), WBC (0.736), and SII (0.656) had good predictive accuracy for massive PE, but platelet count was not a useful predictor (p = 0.089). PESI scores demonstrated a strong association with PE severity, increasing stepwise from non‑massive to massive PE (78.6 ± 37.4 vs. 84.2 ± 32.9 vs. 136.5 ± 54.9; p < 0.001).

Conclusion

Elevated SII, neutrophil count, and WBC count were significantly associated with massive PE and adverse outcomes. These markers offer value for early risk stratification and may enhance clinical decision-making in the management of APE.

## Introduction

Acute pulmonary embolism (APE) is a life-threatening cardiovascular emergency caused by thrombotic obstruction of pulmonary arteries, most commonly originating from deep vein thrombosis. It is a major global cause of cardiovascular mortality, requiring rapid diagnosis and risk stratification for management [1]. APE presents with a wide clinical spectrum, from asymptomatic to sudden cardiac arrest, making early assessment essential to improve outcomes [1,2].

Risk stratification tools, such as the Pulmonary Embolism Severity Index (PESI) [3], and imaging modalities, such as computed tomography pulmonary angiography (CTPA), are widely used in clinical practice. However, these may be limited by cost, availability, and time, especially in resource-constrained settings [2,4]. Consequently, blood-based biomarkers are gaining attention due to their accessibility, low cost, and potential prognostic value. Among these, the systemic immune‑inflammatory index (SII), calculated as platelet count × neutrophil/lymphocyte count, has emerged as a promising indicator of inflammatory and thrombotic activities.

Elmeazawy et al. demonstrated that SII and systemic inflammatory response index (SIRI) effectively distinguished severe necrotizing pneumonia from milder diseases in children, highlighting their diagnostic utility for inflammatory conditions [5]. Gok and Kurtul reported that SII increased with disease severity and independently predicted massive pulmonary embolism (PE), outperforming traditional biomarkers [6]. Haba et al. similarly reported that elevated neutrophil-to-lymphocyte ratio (NLR), platelet-to-lymphocyte ratio (PLR), SIRI, and SII were associated with high-risk APE, with an SII > 1086.55 independently predicting severe disease [7]. Suwadi et al. reinforced these findings in a systematic review, concluding that higher SII levels were consistently correlated with worse outcomes [8]. Uslu et al. demonstrated that SII and SIRI were elevated in intermediate-risk APE and associated with right ventricular (RV) dysfunction [9].

However, despite growing evidence, the prognostic role of SII in Middle Eastern populations remains underexplored. Thus, this study aimed to evaluate the SII as a predictor of APE severity among patients treated at King Fahad Specialist Hospital in the Qassim province in Saudi Arabia.

## Materials and methods

This was a retrospective study involving patients diagnosed with PE at King Fahad Specialist Hospital, Buraydah, Qassim, Saudi Arabia, from 2022 to 2025. The study was approved by the Regional Research Ethics Committee, Al-Qassem Region, Ministry of Health, Kingdom of Saudi Arabia (approval number: 7220/46/607).

Study population

The patients included in the study were admitted with PE diagnosed using CTPA. Patients with conditions affecting blood cell counts, such as sepsis, infections, chronic inflammatory conditions, hematological malignancies, or other hematological causes such as aplastic and megaloblastic anemia, and those with incomplete hospital records were excluded.

Data collection

Data were extracted from the patient records and entered into an Excel sheet (Microsoft Corporation, Redmond, Washington, United States) for analysis. Data included demographics (age and sex) and chronic diseases (diabetes mellitus, hypertension, ischemic heart disease, heart failure, chronic lung disease, stroke, atrial fibrillation, anti-phospholipid syndrome, and other thrombophilia such as antithrombin III deficiency, protein C deficiency, and protein S deficiency). History of malignancy and smoking was noted.

Clinical assessments

Clinical features such as cough, dyspnea, hemoptysis, chest pain, and altered mental status were assessed. Vital status and oxygen saturation were measured on admission. Laboratory results (complete blood counts with differential and troponin levels), echocardiography, and CTPA were collected. SII was calculated as \( \text{Platelet count} \times \left(\frac{\text{Neutrophil count}}{\text{Lymphocyte count}}\right) \).

PE severity was analyzed using the PESI score, which was calculated at the time of diagnosis using the validated scoring system, which assigns weighted points based on age, sex, comorbidities (malignancy, heart failure, and chronic lung disease), and clinical parameters including pulse rate, systolic blood pressure, respiratory rate, temperature, mental status, and oxygen saturation.

Stratification and definitions

Patients were stratified into five risk classes according to the total score: Class I (very low risk, ≤65 points), Class II (low risk, 66-85 points), Class III (intermediate risk, 86-105 points), Class IV (high risk, 106-125 points), and Class V (very high risk, >125 points), with higher classes indicating increased predicted 30-day mortality risk in acute pulmonary embolism. In addition, severity was categorized as massive, submassive, or non-massive PE based on hemodynamic status, RV strain, and cardiac biomarkers. Non-massive PE included hemodynamically stable patients without RV strain or troponin elevation; submassive PE included stable patients with RV strain and/or elevated troponin above the upper limit of troponin I >0.04 ng/mL, while massive PE was defined by hemodynamic instability, including systolic blood pressure <90 mmHg, vasopressor requirement, or cardiac arrest.

Statistical analysis

Data analysis was conducted using IBM SPSS Statistics for Windows, version 26 (IBM Corp., Armonk, New York, United States). Categorical variables were reported as numbers and percentages. Continuous variables were reported as means and standard deviations, as well as medians with minimum and maximum values. PE severity was compared with the patients' demographic and clinical characteristics using Fisher's exact test, independent sample t-test, one-way analysis of variance, and Kruskal-Wallis test. The association between mortality rates and patient demographic and clinical characteristics was evaluated using Fisher's exact test and an independent-samples t-test. Normality test has been performed using the Shapiro-Wilk test. Receiver operating characteristic (ROC) analysis was performed for SII, neutrophil, and platelet counts to determine cutoffs for significance in massive APE. Statistical significance was set at p < 0.05.

## Results

As shown in Table 1, the study included 166 patients with a mean age of 57.1 years; 93 patients (56%) were female. Comparison of PE severity with age and sex showed that neither age nor sex was significantly associated with PE severity (p > 0.05).

**Table 1:** Demographic characteristics of the patients (N=166) ‡ P-value has been calculated using one-way analysis of variance test; § P-value has been calculated using Fisher's Exact test. PE: pulmonary embolism

Study variables		Overall	PE Severity			F-test	p-value
			Massive (n = 25)	Submassive (n = 66)	Non-massive (n = 75)		
Age (years), mean ± SD		57.1 ± 19.4	60.9 ± 21.3	56.7 ± 18.1	56.2 ± 19.9	0.582	0.560 ^‡^
Sex, n (%)	Male	73 (44.0%)	08 (32.0%)	28 (42.4%)	37 (49.3%)	NA	0.302 ^§^
	Female	93 (56.0%)	17 (68.0%)	38 (57.6%)	38 (50.7%)		

Table 2 shows that diabetes (n=61, 36.7%) and hypertension (n=73, 44%) were the most common comorbidities. Other conditions, such as ischemic heart disease (n=16, 9.6%), heart failure (n=21, 12.7%), and chronic kidney disease (n=17, 10.2%), were less frequent. Only 12 (7.2%) patients smoked. Echocardiograms showed a normal ejection fraction in 118 (71.1%) patients, while 84 (50.6%) had RV strain. The mortality rate was 27 (16.3%). When comparing across PE severity groups, most demographic and comorbidity variables, including diabetes, hypertension, ischemic heart disease, heart failure, chronic kidney disease, atrial fibrillation, chronic lung disease, and cancer, did not differ significantly (all p > 0.05). However, several variables showed significant associations with PE severity. A history of prior stroke was more common in massive PE (n=8, 32.0%) than in submassive PE (n=4, 6.1%) or non‑massive PE (n=12, 16.0%) (p = 0.007). Smoking was significantly higher in the submassive group (p = 0.035). Bradycardia was significantly more common in massive PE (n=4, 16.0%) (p = 0.005). Echocardiographic findings differed significantly (p < 0.001), with massive PE showing higher rates of reduced ejection fraction. RV strain was highly prevalent in both massive PE (n=22, 88.0%) and submassive PE (n=61, 92.4%). Mortality was significantly higher in massive PE (32.0%) than in submassive (10.6%) or non‑massive PE (16.0%) (p = 0.047). Overall, the data show that neurological impairment, hemodynamic compromise, bradycardia, abnormal echocardiographic findings, and RV strain are strongly associated with PE severity, while most baseline comorbidities do not significantly differentiate severity categories.

**Table 2:** Clinical characteristics and outcome of the patients § p-value has been calculated using Fisher's Exact test; ** Significant at p < 0.05 level. PE: pulmonary embolism; Afib: atrial fibrillation; AMS: altered mental status; RV: right ventricle; EF: ejection fraction; APS: antiphospholipid syndrome

Variables		Overall (N = 166), n (%)	PE Severity, n (%)			
			Massive (n = 25)	Submassive (n = 66)	Non-massive (n = 75)	p-value ^§^
Diabetes	No	105 (63.3%)	15 (60.0%)	42 (63.6%)	48 (64.0%)	0.934
	Yes	61 (36.7%)	10 (40.0%)	24 (36.4%)	27 (36.0%)	
Hypertension	No	93 (56.0%)	16 (64.0%)	34 (51.5%)	43 (57.3%)	0.537
	Yes	73 (44.0%)	09 (36.0%)	32 (48.5%)	32 (42.7%)	
Ischemic heart disease	No	150 (90.4%)	22 (88.0%)	61 (92.4%)	67 (89.3%)	0.728
	Yes	16 (09.6%)	03 (12.0%)	05 (07.6%)	08 (10.7%)	
Heart failure	No	145 (87.3%)	21 (84.0%)	59 (89.4%)	65 (86.7%)	0.701
	Yes	21 (12.7%)	04 (16.0%)	07 (10.6%)	10 (13.3%)	
Chronic kidney disease	No	149 (89.8%)	22 (88.0%)	57 (86.4%)	70 (93.3%)	0.363
	Yes	17 (10.2%)	03 (12.0%)	09 (13.6%)	05 (06.7%)	
Old stroke	No	142 (85.5%)	17 (68.0%)	62 (93.9%)	63 (84.0%)	0.007 **
	Yes	24 (14.5%)	08 (32.0%)	04 (06.1%)	12 (16.0%)	
Afib	No	153 (92.2%)	21 (84.0%)	62 (93.9%)	70 (93.3%)	0.306
	Yes	13 (07.8%)	04 (16.0%)	04 (06.1%)	05 (06.7%)	
APS	No	163 (98.2%)	25 (100%)	66 (100%)	72 (96.0%)	0.283
	Yes	03 (01.8%)	0 (0%)	0 (0%)	03 (04.0%)	
Chronic lung disease	No	148 (89.2%)	23 (92.0%)	61 (92.4%)	64 (85.3%)	0.387
	Yes	18 (10.8%)	02 (08.0%)	05 (07.6%)	11 (14.7%)	
History of cancer	No	151 (91.0%)	24 (96.0%)	61 (92.4%)	66 (88.0%)	0.504
	Yes	15 (09.0%)	01 (04.0%)	05 (07.6%)	09 (12.0%)	
Smoker	No	154 (92.8%)	25 (100.0%)	57 (86.4%)	72 (96.0%)	0.035 **
	Yes	12 (07.2%)	0	09 (13.6%)	03 (04.0%)	
Dyspnea	No	11 (06.6%)	01 (04.0%)	07 (10.6%)	03 (04.0%)	0.275
	Yes	155 (93.4%)	24 (96.0%)	59 (89.4%)	72 (96.0%)	
Cough	No	127 (76.5%)	16 (64.0%)	52 (78.8%)	59 (78.7%)	0.278
	Yes	39 (23.5%)	09 (36.0%)	14 (21.2%)	16 (21.3%)	
Hemoptysis	No	148 (89.2%)	22 (88.0%)	62 (93.9%)	64 (85.3%)	0.252
	Yes	18 (10.8%)	03 (12.0%)	04 (06.1%)	11 (14.7%)	
Chest pain	No	75 (45.2%)	10 (40.0%)	23 (34.8%)	42 (56.0%)	0.035 **
	Yes	91 (54.8%)	15 (60.0%)	43 (65.2%)	33 (44.0%)	
AMS	No	139 (83.7%)	10 (40.0%)	61 (92.4%)	68 (90.7%)	<0.001 **
	Yes	27 (16.3%)	15 (60.0%)	05 (07.6%)	07 (09.3%)	
Bradycardia	No	160 (96.4%)	21 (84.0%)	65 (98.5%)	74 (98.7%)	0.005 **
	Yes	06 (03.6%)	04 (16.0%)	01 (01.5%)	01 (01.3%)	
Hemodynamic instability	No	141 (84.9%)	0 (0%)	66 (100%)	75 (100%)	<0.001 **
	Yes	25 (15.1%)	25 (100.0%)	0 (0%)	0 (0%)	
Echo	Not done	12 (07.2%)	05 (20.0%)	02 (03.0%)	05 (06.7%)	<0.001 **
	Normal EF	118 (71.1%)	08 (32.0%)	48 (72.7%)	62 (82.7%)	
	EF <40%	09 (05.4%)	04 (16.0%)	05 (07.6%)	0 (0%)	
	EF 40 – 49%	27 (16.3%)	08 (32.0%)	11 (16.7%)	08 (10.7%)	
RV strain	No	82 (49.4%)	03 (12.0%)	05 (07.6%)	75 (100%)	<0.001 **
	Yes	84 (50.6%)	22 (88.0%)	61 (92.4%)	0 (0%)	
Mortality	No	139 (83.7%)	17 (68.0%)	59 (89.4%)	63 (84.0%)	0.047 **
	Yes	27 (16.3%)	08 (32.0%)	07 (10.6%)	12 (16.0%)	

Table 3 shows mean systolic and diastolic blood pressures were 121.7 ± 22.8 mmHg and 74.2 ± 13.4 mmHg, respectively, with a mean heart rate of 98.5 ± 20.9 bpm, and the respiratory rate averaged 20.6 ± 6.08 breaths/min. Laboratory parameters showed a mean white blood cell (WBC) count of 10.9 ± 5.05 × 10⁹/L, neutrophils at 7.83 ± 4.61 × 10⁹/L, lymphocytes at 2.04 ± 1.16 × 10⁹/L, and eosinophils at 0.16 ± 0.56 × 10⁹/L. Hemoglobin averaged 12.7 ± 2.39 g/dL, platelet count 261.6 ± 121.5 × 10⁹/L, and troponin 0.21 ± 1.12 ng/mL. The SII was 1317.6 ± 1166. Several variables showed significant differences between PE severity groups. Patients with massive PE had much lower systolic (85.8 ± 18.9 mmHg) and diastolic blood pressure (55.8 ± 15.6 mmHg) than those with submassive or non-massive PE (both p < 0.001), indicating severe hemodynamic compromise. Their heart rates were higher (106.3 ± 34.9 beats per minute, p = 0.003). Inflammatory markers, such as WBC (14.6 ± 8.07) and neutrophils (11.3 ± 7.27), were higher in massive PE than in other groups (both p < 0.001). Patients with massive PE had lower hemoglobin levels (11.6 ± 2.22 g/dL, p = 0.037). Platelet counts were highest in those with non-massive PE (p = 0.009). Troponin levels were higher in patients with massive PE (1.28 ± 3.02 ng/mL) than in those with submassive (0.13 ± 0.30 ng/mL) or non-massive PE (0.01 ± 0.03 ng/mL) (p < 0.001). SII was highest in those with massive PE (p = 0.022). These results show that massive PE is associated with severe hemodynamic instability, greater inflammation, higher levels of cardiac injury markers, and lower hemoglobin, distinguishing it from submassive and non-massive PE.

**Table 3:** Metabolic and laboratory characteristics of the patients in relation to PE severity ^§^ p-value has been calculated using a one-way analysis of variance test; ^**^ Significant at p < 0.05 level SBP: systolic blood pressure; DBP: diastolic blood pressure; HR: heart rate; RR: respiratory rate; WBC: white blood cell; Eos: eosinophils; Hb: hemoglobin; TROP: troponin; SII: systemic immune‑Inflammatory index

Variables	Overall (N = 166), mean ± SD	PE Severity, mean ± SD			F-test	p-value ^§^
		Massive (n = 25)	Submassive (n = 66)	Non-massive (n = 75)		
SBP (mmHg)	121.7 ± 22.8	85.8 ± 18.9	127.2 ± 19.7	127.5 ± 15.8	51.257	<0.001 **
DBP (mmHg)	74.2 ± 13.4	55.8 ± 15.6	77.3 ± 10.6	76.9 ± 10.4	33.911	<0.001 **
HR (beats/minute)	98.5 ± 20.9	106.3 ± 34.9	102.6 ± 17.6	92.6 ± 16.4	6.139	0.003 **
RR (breaths/min)	20.6 ± 6.08	23.3 ± 10.0	20.6 ± 6.14	19.9 ± 4.82	1.747	0.178
WBC (× 10⁹/L)	10.9 ± 5.05	14.6 ± 8.07	10.7 ± 3.89	9.97 ± 4.11	8.632	<0.001 **
Neutrophil (× 10⁹/L)	7.83 ± 4.61	11.3 ± 7.27	7.62 ± 3.49	6.85 ± 3.80	9.773	<0.001 **
Lymph (× 10⁹/L)	2.04 ± 1.16	1.96 ± 1.62	2.10 ± 1.21	2.01 ± 0.92	0.185	0.831
Eos (× 10⁹/L)	0.16 ± 0.56	0.09 ± 0.15	0.22 ± 0.87	0.13 ± 0.16	0.617	0.541
Hb (g/dL)	12.7 ± 2.39	11.6 ± 2.22	12.9 ± 2.47	12.9 ± 2.32	3.367	0.037 **
Platelet (× 10⁹/L)	261.6 ± 121.5	246.2 ± 114.4	232.1 ± 116.8	292.6 ± 111.1	4.806	0.009 **
TROP (ng/mL)	0.21 ± 1.12	1.28 ± 3.02	0.13 ± 0.30	0.01 ± 0.03	9.622	<0.001 **
SII	1317.6 ± 1166	1890.6 ± 1384	1149.5 ± 981.4	1273.9 ± 1166	3.217	0.022

The multivariate logistic regression model identified several independent predictors of massive PE after adjusting for sex, diabetes, and hypertension (Table 4). RV strain was the strongest predictor. Patients with RV strain were 44 times more likely to experience massive PE (adjusted odds ratio (AOR) 44.135, 95%CI, 15.394-126.5; p < 0.001), highlighting its significance in diagnosis and prognosis. Although an association with mortality was observed, it was not significant (aOR 0.461, 95%CI 0.182-1.169; p = 0.103). Overall, these results indicated that RV strain was the only key independent predictor of massive PE.

**Table 4:** Multivariate logistic regression model showing independent associations between clinical variables and massive PE after adjustment for sex, diabetes, and hypertension Adjusted for sex, diabetes, and hypertension; ** Significant at p < 0.05 level. PE: pulmonary embolism; aOR: adjusted odds ratio; RV: right ventricular

Factor		aOR	95% CI	p-value
RV strain	No	Ref	-	<0.001 **
	Yes	44.135	15.394–126.5	
Mortality	No	Ref	-	0.103
	Yes	0.461	0.182–1.169	

**Figure 1:**
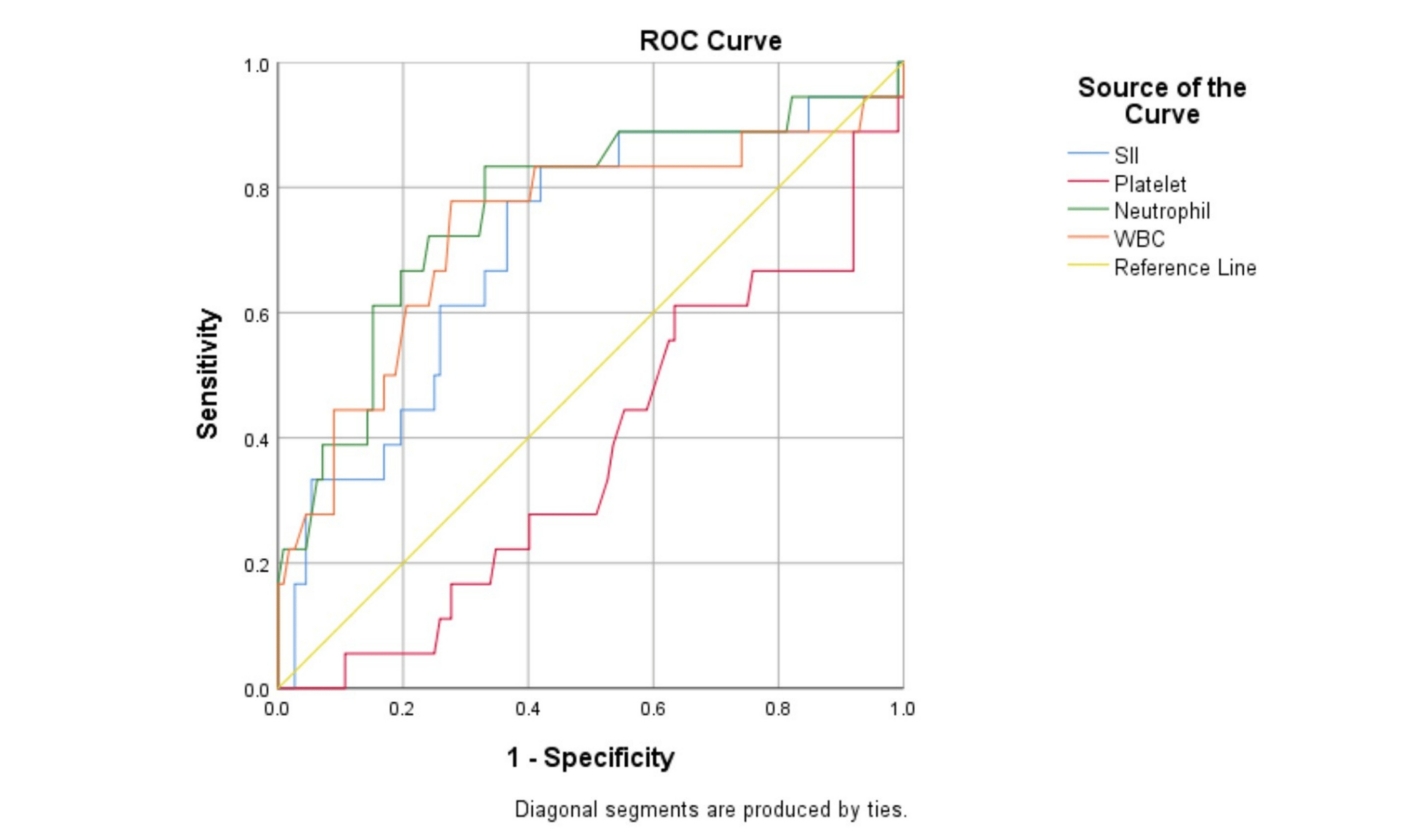
ROC curves of SII, neutrophil count, and platelet count in predicting massive acute pulmonary embolism. SII: systemic immune-inflammation index; ROC: receiver operating characteristic

Table 5 and Figure 1 show that the SII, neutrophil count, platelet count, and WBC count predict massive PE. ROC analysis showed that neutrophil count had the highest accuracy (area under the curve (AUC): 0.766; p < 0.001). WBC count followed (AUC: 0.736; p = 0.001) and SII (AUC: 0.656; p = 0.013). The optimal SII cutoff value for predicting massive PE was 372, with 88% sensitivity and 15% specificity. Platelet count had little predictive value (AUC: 0.375; p = 0.089) and was not useful.

**Table 5:** Statistics results for the AUC of SII, platelets, and neutrophils in predicting massive acute PE ** Significant at p < 0.05 level. SII: systemic immune‑inflammatory index; PE: pulmonary embolism; WBC: white blood cell; AUC: area under the curve

Variable	Cutoff point	Sensitivity	Specificity	AUC	Std. Error	p-value	Asymptotic 95% CI	
							Lower Bound	Upper Bound
SII	372	88.0%	15.0%	0.656	0.068	0.013 **	0.528	0.783
Platelet (× 10⁹/L)	216	61.1%	63.4%	0.375	0.070	0.089	0.239	0.511
Neutrophil (× 10⁹/L)	5.75	88.9%	58.0%	0.766	0.068	<0.001 **	0.633	0.899
WBC (× 10⁹/L)	5.90	88.9%	89.3%	0.736	0.075	0.001 **	0.590	0.882

Table 6 shows that the PESI score demonstrated a strong overall association with PE severity, with a mean score of 89.6 ± 43.5 across all patients. When stratified by severity, PESI scores increased stepwise, with patients with massive PE exhibiting the highest scores (136.5 ± 54.9) compared with submassive (84.2 ± 32.9) and non‑massive PE (78.6 ± 37.4), indicating significantly greater clinical compromise (p < 0.001). A similar gradient was observed across PESI classes: very‑low‑ and low‑risk categories (Classes I-II) were predominantly observed in non‑massive PE, whereas more than half of patients with massive PE were in the very‑high‑risk Class V (56.0%), further confirming the strong correlation between higher PESI categories and increasing PE severity (p < 0.001).

**Table 6:** Association between PESI and PE severity ^§^ P-value has been calculated using a one-way analysis of variance test; ^‡^ P-value has been calculated using Fisher's Exact test; ** Significant at p < 0.05 level PESI: pulmonary embolism severity index; PE: pulmonary embolism; NA: not applicable.

Variables	Overall (N = 166)	PE Severity			F-test	P-value
		Massive (n = 25)	Submassive (n = 66)	Non-massive (n = 75)		
PESI score, mean ± SD ^§^	89.6 ± 43.5	136.5 ± 54.9	84.2 ± 32.9	78.6 ± 37.4	21.916	<0.001 **
PESI class, n (%) ^‡^						
Class I: Very low risk	52 (31.3%)	2 (8.0%)	18 (27.3%)	32 (42.7%)	NA	<0.001 **
Class II: Low risk	38 (22.9%)	4 (16.0%)	21 (31.8%)	13 (17.3%)		
Class III: Intermediate risk	32 (19.3%)	2 (8.0%)	13 (19.7%)	17 (22.7%)		
Class IV: High risk	13 (7.8%)	3 (12.0%)	7 (10.6%)	3 (4.0%)		
Class V: Very high risk	31 (18.7%)	14 (56.0%)	7 (10.6%)	10 (13.3%)		

## Discussion

This study investigated the prognostic value of the SSI for predicting APE severity. These findings demonstrate that inflammatory and hemodynamic markers can effectively differentiate massive from submassive and non‑massive PE, supporting the role of thromboinflammatory pathways in disease progression. Baseline characteristics such as age, sex, and comorbidities did not significantly differ across the severity groups, suggesting acute physiological responses may be more influential than chronic factors. This aligns with the findings of Becattini et al., who reported that clinical deterioration and RV dysfunction are stronger predictors of mortality than demographic variables [10].

Patients with massive PE exhibit higher rates of altered mental status, bradycardia, and hemodynamic instability, which are hallmark features of high-risk PE, as described in the 2019 European Society of Cardiology (ESC) guidelines [1]. Chest pain was most common in submassive PE, consistent with observations by Stein et al., linking pleuritic symptoms to embolic distribution [11]. Echocardiographic findings distinguished the severity groups, with massive PE showing reduced ejection fraction and marked RV strain. This supports the study by Karahan and Okuyan, emphasizing the prognostic importance of cardiac dysfunction in PE [12].

Laboratory parameters showed a severity gradient, with elevated WBC and neutrophil counts and reduced hemoglobin levels in patients with massive PE, reflecting heightened inflammation and impaired oxygen-carrying capacity. Peng et al. similarly reported that elevated neutrophil levels correlated with greater pulmonary arterial obstruction [13]. Haba et al. reported that the NLR and SII predicted high-risk APE, consistent with our findings [7].

The SII was significantly higher in massive PE, consistent with those reported by Özdemir et al. [14] and Duyan et al. [15], showing that elevated SII correlates with PE severity and adverse outcomes. Ösken and Çam showed that SII predicts long‑term outcomes, suggesting utility beyond the acute phase [16]. In our ROC analysis, the neutrophil count (AUC 0.766), WBC count (AUC 0.736), and SII (AUC 0.656) demonstrated good predictive performance. Although the SII demonstrated high sensitivity in identifying massive PE, its specificity was low (15%), indicating a substantial false-positive rate. This suggests the SII is better as a marker to flag patients with heightened thromboinflammatory activity rather than as a tool for excluding lower-risk patients. In contrast, neutrophil and WBC counts showed a stronger overall diagnostic performance, with higher AUC values and more balanced sensitivity-specificity profiles. Therefore, the SII should be interpreted with conventional inflammatory markers, not used alone.

Platelet count did not predict outcomes, aligning with Phan et al., who reported that platelet-based ratios are less reliable than neutrophil-based indices in PE [17]. Troponin levels showed the clearest severity gradient, highlighting the role of myocardial injury in identifying high-risk patients.

In addition, the prognostic performance of the PESI score in our cohort closely mirrors international findings. PESI scores increased stepwise from non‑massive to massive PE, with massive cases demonstrating markedly higher mean scores (136.5 ± 54.9) and a predominance in the very‑high‑risk Class V category (56%). This gradient aligns with the 2019 ESC guidelines, which emphasize PESI as a validated tool for early risk stratification and mortality prediction in acute PE [1]. Similarly, Chan et al. demonstrated the strong reproducibility of PESI and its ability to identify high-risk patients [2], whereas Becattini et al. linked higher PESI classes with RV dysfunction and adverse outcomes [10]. The alignment between our findings and those of these established studies reinforces the robustness of PESI for identifying clinically unstable patients, even in the Middle Eastern population. The concordance between elevated PESI class, hemodynamic compromise, and RV strain in our cohort underscores its value as an integrated severity assessment tool.

Overall, integrating inflammatory markers such as the SII with hemodynamic and echocardiographic parameters enhances early risk stratification in APE. Routine SII monitoring may help identify high-risk patients earlier and guide targeted management.

Study limitations

However, some limitations should be considered when generalizing these results. First, as the study was conducted at only one hospital, the findings may not apply elsewhere. Second, although the SII, WBC, and neutrophil counts were associated with PE severity, the SII had low specificity, with only 15% at the best cutoff, suggesting it may not be a reliable standalone prognostic marker. Third, we did not compare the SII with other well-known biomarkers, such as D-dimer, N-terminal pro-B-type natriuretic peptide, or lactate, which could have provided a complete picture of its prognostic value. Finally, as this was an observational study, we cannot conclude causation, and more multicenter studies are needed to confirm these results and find better cutoff values.

## Conclusions

This study supports the finding that the SII has a prognostic value in identifying patients with massive PE. Massive PE is characterized by hemodynamic compromise, neurological impairment, elevated inflammatory marker levels, and marked RV strain. Among the evaluated biomarkers, neutrophil count, WBC count, and SII demonstrated the strongest predictive performance, whereas platelet count alone was less informative. Multivariate analysis identified RV strain, low systolic and diastolic blood pressures, tachycardia, and elevated platelet levels as independent predictors of massive PE. Although SII was significantly higher in patients with massive PE, its low specificity suggests it as a marker rather than a reliable tool for excluding lower-risk patients. Neutrophil and WBC counts showed better diagnostic accuracy and should be prioritized for precision. PESI scores and classes demonstrated a strong stepwise association with PE severity, which is consistent with international validation studies. The predominance of patients in Class V among those with massive PE highlights the continued relevance of PESI as a rapid and reliable tool for early risk stratification in diverse clinical settings. Overall, integrating inflammatory markers with clinical findings and echocardiographic assessments enhanced early risk stratification. Incorporating the SII in a multimodal evaluation may help identify high-risk patients earlier and support more targeted management strategies.
